# Identification and Characterization of an *Arabidopsis thaliana* Mutant *lbt* With High Tolerance to Boron Deficiency

**DOI:** 10.3389/fpls.2018.00736

**Published:** 2018-06-04

**Authors:** Zexun Huai, Lishun Peng, Sheliang Wang, Hua Zhao, Lei Shi, Fangsen Xu

**Affiliations:** ^1^National Key Laboratory of Crop Genetic Improvement, Microelement Research Center, Huazhong Agricultural University, Wuhan, China; ^2^Institute of Tropical Bioscience and Biotechnology, Chinese Academy of Tropical Agricultural Sciences, Haikou, China

**Keywords:** *Arabidopsis thaliana*, low-boron, tolerance, *lbt* mutant, genetic analysis, starch accumulation

## Abstract

Boron (B) is an essential micronutrient of plants. In the present study, we characterized an *Arabidopsis* mutant *lbt* with significant **l**ow**-b**oron **t**olerance that was identified based on our previous mapping of QTL for B efficiency in *Arabidopsis*. Multiple nutrient-deficiency analyses point out that *lbt* mutant is insensitive to only B-limitation stress. Compared with wild-type Col-0, the fresh weight, leaf area, root length and root elongation rate of *lbt* mutant were significantly improved under B deficiency during vegetative growth. *lbt* mutant also showed the improvements in plant height, branches and inflorescences compared with Col-0 during the reproductive stage under B limitation. Ultrastructure analysis of the leaves showed that starch accumulation in *lbt* mutant was significantly diminished compared with Col-0. Furthermore, there were no significant differences in the expression of transporter-related genes and B concentrations between Col-0 and *lbt* mutant under both normal B and low-B conditions. These results suggest that *lbt* mutant has a lower B demand than Col-0. Genetic analysis suggests that the low-B tolerant phenotype of *lbt* mutant is under the control of a monogenic recessive gene. Based on the high-density SNP linkage genetic map, only one QTL for low-B tolerance was mapped on chromosome 4 between 10.4 and 14.8 Mb. No any reported B-relative genes exist in the QTL interval, suggesting that a gene with unknown function controls the tolerance of *lbt* to B limitation. Taken together, *lbt* is a low-B tolerant mutant that does not depend on the uptake or transport of B and is controlled by a monogenic recessive gene mapped on chromosome 4, and cloning and functional analysis of *LBT* gene are expected to reveal novel mechanisms for plant resistance to B deficiency.

## Introduction

Boron (B) is essential for the development and growth of higher plants (Warington, 1923). Considerable yield reduction in crop plants due to B deficiency is a worldwide agricultural problem (Shorrocks, 1997). Typical symptoms of B deficiency are reflected in the brittleness of leaves and inhibition of root elongation (Shorrocks, 1997). B deficiency hampers many physiological and metabolic aspects of plants, including sugar transport, cell wall structure, photosynthesis, starch granule synthesis and the metabolism of indole acetic acid (Herrera-Rodríguez et al., 2010; Li et al., 2015). B plays an important structural role in the cross-linking of cell wall rhamnogalacturonan II (RG-II) to form a stable three-dimensional pectic network which contributes to the mechanical properties of cell wall structure (Funakawa and Miwa, 2015), or vice versa, and many of the effects associated with B deficiency could be the consequence of a disturbed cell wall structure (Camacho-Cristóbal et al., 2015).

In *Arabidopsis thaliana*, genes involved in B uptake and transport have been cloned, and they include the BOR transporter gene family and NIP (NOD26-like intrinsic proteins) channel gene family. BOR1 is an efflux-type transporter for xylem loading in root, which is an important process for shoot response to B deficiency (Takano et al., 2002). *BOR2* is essential for effective RG-II cross linking in the cell wall and root elongation under B limitation (Miwa et al., 2013). *BOR4* is critical for B export from plant roots to prevent the over accumulation of B in root cells (Miwa et al., 2007). Aquaporin channel proteins NIP5;1 and NIP6;1 are required for the efficient influx of B from soils into root cells and the preferential distribution of B into young tissues, respectively (Takano et al., 2006; Tanaka et al., 2008). NIP7;1 is a channel protein with an extremely low intrinsic boric acid transport activity and is expressed principally in developing pollen grains (Li et al., 2011).

*Arabidopsis thaliana* mutant *bor-1* is extremely sensitive to B deficiency, and its shoot growth was more inhibited than root growth, and the main reason for the defects in the *bor1-1* mutant growth is the disability of B transport from root to shoot (Noguchi et al., 1997, 2000; Takano et al., 2001). Under the condition of B deficiency, overexpression lines of the *BOR1* gene in *A. thaliana* wild-type showed increased B transport and shoot growth, which improved the fertility and seed yield of the plants (Miwa et al., 2006). Expression of *NIP5;1* in response to B deficiency is mainly controlled by a conservative AUGUAA (AUG-stop) sequence in the 5′UTR (Tanaka et al., 2016). The polar localization of *NIP5;1* in root was mediated by the phosphorylation of Thr in a conserved three-amino acid (ThrProGly) repeat in its N-terminal region (Wang et al., 2017). Overexpression of the *NIP5;1* gene in the *nip5;1* mutant could improve root elongation, fertility and B uptake under B deficiency conditions (Kato et al., 2009). However, there is few genes to be cloned related to low-B tolerance except NIPs family and the BORs family.

Map-based cloning is one of the most available methods to identify unknown function genes in plants, such as *BOR1* (Takano et al., 2002). Quantitative trait loci (QTL) mapping is an important first step for map-based cloning and illuminating the genetic characteristics of complex traits (Mauricio, 2001). However, the identification of QTL mapping depends on a large of population of individuals and a high-density linkage map, which is laborious and time-consuming to construct (Shen et al., 2018). Next-generation sequencing (NGS) technologies provide a wealth of previously impossible approaches for genetic studies. High-throughput-based whole genome sequencing (WGS) is efficient for SNP discovery and high-density genetic map construction (Xu and Bai, 2015).

One of the QTLs for B efficiency, *AtBE1-2* was mapped to the chromosome 1 of *A. thaliana* (Zeng et al., 2008). Fifty-eight genes in the confidence interval of *AtBE1-2* showed up- or down-regulation under B deficiency based on a transcript profile analysis (Peng et al., 2012). T-DNA insertion mutants for the 58 genes acquired through *A. thaliana* Biological Resource Center (ABRC^1^) were employed to assess low-B tolerance capacity under low-B conditions. There were no significant differences in growth phenotypes under normal B (30 μM) condition between wild-type Col-0 and all mutants (data not shown). However, one homozygous mutant with T-DNA insertion in the exon of At1g78860 (SALK_144144C) showed significantly higher tolerance to low-B (0.1 μM) stress than Col-0, and we named it ‘*lbt* mutant.’ In this study, we phenotypically characterized the mutant with improved growth performance under B limitation in both vegetative and reproductive growth and analyzed its physiological characterizations in response to low-B stress. Furthermore, a major QTL for low-B tolerance was mapped to the region of 10.4–14.8 Mb on chromosome 4.

## Materials and Methods

### Plant Materials and Growth Conditions

*Arabidopsis thaliana* Col-0 was kept in our laboratory stock. Two T-DNA insertion mutant lines of At1g78860 with a Col-0 background, SALK_144144C (*lbt* mutant) and SALK_021225C, were acquired through *A. thaliana* Biological Resource Center (ABRC^1^). Two F_2_ populations were developed and used in the present study. The first F_2_ population was derived from a cross between Col-0 and *lbt* mutant (SALK_144144C) and used to analyze the co-segregation of the low-B tolerance phenotype with the genotype of T-DNA insertion in At1g78860. Another F_2_ mapping population was developed by a cross of *lbt* mutant and Cs1909 line to map the QTL for low-B tolerance of *lbt* mutant. Map-based cloning needs two genotypes with significant differences in target traits and genetic variation in parents to facilitate the development of molecular markers for the construction of a linkage genetic map. Col-0 and *lbt* mutant have a similar genetic background, so it is difficult to find additional polymorphism molecular markers from the two genotypes for the construction of a linkage map. Landsberg *erecta* (L*er*) showed tolerance to low-B stress (identified in our previous study) and was not suitable as a parent with the low-B tolerance *lbt* mutant to develop a F_2_ population. Cs1909 is a low-B sensitive line selected from a recombinant inbred line (RIL) population that was developed by a cross between L*er* and Columbia-4 (Col-4).

Seeds of the plants described above were surface-sterilized using 1% (w/v) NaClO for 10 min and rinsed in ultrapure water. The seeds were then planted on solid medium [1.51 mM NaH_2_PO_4_, 0.26 mM Na_2_HPO_4_, 1.5 mM MgSO_4_, 2.0 mM Ca(NO_3_)_2_, 3.0 mM KNO_3_, 10.3 μM MnSO_4_, 1.0 μM ZnSO_4_, 1.0 μM CuSO_4_, 130 nM CoCl_2_, 24 nM (NH_4_)_6_Mo_7_O_24_, and 50 μM FeNa-EDTA] (Fujiwara et al., 1992) containing 1% sucrose and 1% Gellan Gum supplemented with 30 μM or 0.1 μMB, respectively. For other element stress treatments, plants were cultivated in a hydroponic culture system with Hoagland and Arnon solution [5 mM KNO_3_, 1 mM KH_2_PO_4_, 2 mM MgSO_4_, 5 mM Ca(NO_3_)_2_, 50 μM FeNa-EDTA, 9 μM MnCl_2_, 300 nM CuSO_4_, 800 nM ZnSO_4_, and 370 nM Na_2_MoO_4_] (Hoagland and Arnon, 1950) for 10 days and then transferred into various stress conditions for 10 additional days of growth. These stress conditions included -B (0.1 μM), -N (2 μM), -P (2 μM), -K (2 μM), -Fe (0.1 μM), and ++B (3 mM). For -N treatment, KNO_3_ and Ca(NO_3_)_2_ were replaced by 2.5 mM K_2_SO_4_ and 5 mM CaSO_4_, respectively. For -K treatment, KNO_3_ and KH_2_PO_4_ were replaced by 2.5 mM Ca(NO_3_)_2_ and 1 mM NaH_2_PO_4_, respectively. For -P treatment, KH_2_PO_4_ was replaced by 0.5 mM K_2_SO_4_ (Qin et al., 2012). All plants were cultivated with a temperature regime of 22/20°C (day/night) and a photoperiod of 16/8 h (day/night) with a light intensity of 300–320 μmol⋅m^-2^⋅s^-1^. For the phenotypic characterization of reproductive growth, Col-0 and *lbt* mutant were grown on solid vertical pates with 30 μMB for 10 days and then transferred to the hydroponic culture system with various B conditions (0.5, 1, 3, 10, or 30 μM) for 25–35 days of additional growth.

### Co-segregation Analysis

To verify whether the low-B tolerance of *lbt* mutant was caused by At1g78860, we analyzed the co-segregation of the low-B tolerance phenotype with the T-DNA insertion genotype of At1g78860. Low-B tolerance individuals in the F_2_ population from the cross between Col-0 and *lbt* mutant were used to identify the low-B tolerant genotype by PCR with three primers. The forward/reverse primers of At1g78860 corresponding to *A. thaliana* genomic sequences flanking the T-DNA insertion and the left border primer of the T-DNA insertion (T-DNA primer) corresponding to the sequence of the T-DNA fragment were designed for PCR analysis used and listed in **Table 1**. If all the low-B tolerance individuals have the homozygous T-DNA insertion genotype (*lbt* mutant genotype), it suggests that the phenotype is co-segregated with T-DNA insertion and that the T-DNA insertion (At1g78860) is relevant for the phenotype of low-B tolerance. Otherwise, any these individuals without the T-DNA insertion of At1g78860 can rule out the co-segregation.

**Table 1:** Primer sequences.

	Primer name	Forward (5′–3′)	Reverse (5′–3′)
Primer for RT-qPCR	*BOR1*	AATCTCGCAGCGGAAACG	TGGAGTCGAACTTGAACTTGTC
	*BOR2*	CATCTCGCAGTACCGGAAGCT	AGCCTTGGACTCATCTCACCT
	*BOR3*	CATTCAATCTCAAACCGGGAAGG	TTCAAGCTGCTCACTTTCCTTA
	*BOR4*	GGAACTGTCTTTCCGGTCGAA	CTTGGGATAAATCTGGTTGCCT
	*NIP5;1*	CACCGATTTTCCCTCTCCTGAT	GCATGCAGCGTTACCGATTA
	*NIP6;1*	GGCAATGGTTACAGCCGGAT	GGAGCTGAGACGCTTATTGGTT
	*NIP7;1*	CATCTCTGGCGCCCATCT	CCGCCAAAGACAGCGAAA
	*Actin*	GTTCCAGCCCTCGTTTGTG	CAAGTGCTGTGATTTCTTTGCTC
	*UBQ5*	GTGGTGCTAAGAAGAGGAAGA	TCAAGCTTCAACTCCTTCTTT
Primer for co-segregation analysis	At1g78860	GGCTTGTCATGAGAAGCAAAG	ACACATGGAAGGTGTCGACTC
	T-DNA	ATTTTGCCGATTTCGGAAC	

### Characterization of the Growth Parameters of Plants

Seven-day-old Col-0 and *lbt* mutant grown on the solid plates with 30 or 0.1 μMB were harvested to measure the shoot fresh weight and imaged to calculate primary root length and lateral root number. Images of 11-day-old plants were taken to calculate the leaf area. The root elongation rate was measured based on the everyday images of plants from 5- to 11-day-olds, the root elongation of each day was measured according to the root length on 1 day minus that on the previous day. ImageJ software was used in this determination^2^.

### Transmission Electron Microscopy and Stereoscopic Microscopy

The root tip distance of 7-day-old seedlings was examined using an Olympus SZX16 stereoscopic microscope (Olympus, Tokyo, Japan). The distance from the root tip to the first appearance of root hairs was quantified using ImageJ^2^. Juvenile leaves of 11-day-old Col-0 and *lbt* mutant grown on solid plates with 30 or 0.1 μMB were fixed in 0.1 M potassium phosphate (pH 6.8) with 2.5% glutaraldehyde at 10 a.m. Then, the samples were dehydrated in a series of ethanol [30, 50, 70, 80, 90, 95, and 100% (v/v) ethanol/water] and propylene oxide for dehydration and embedded in epoxy resin. Ultramicrotome (UC6/FC6; Leica, Germany) was used to prepare ultra-thin sections (0.5–1.0 μm) (Zhang et al., 2017). Specimens of the ultrastructure were observed using transmission electron microscopy (TEM) (H-7650; Hitachi, Tokyo, Japan).

### Real-Time Quantitative PCR

Nine-day-old Col-0 and *lbt* mutant grown vertically on solid plates with 30 μMB were transferred to 30 or 0.1 μMB medium for 48 h and then harvested for total RNA extraction by the Eastep^®^ Super Total RNA Extraction Kit (Promega, Madison, WI, United States). cDNA was prepared by reverse transcription with M-MLV reverse transcriptase (Promega, Madison, WI, United States) and oligo (dT)_15_ primers (Promega, Madison, WI, United States) according to the manufacturer’s protocol. RT-qPCR assays for the detection of the relative gene expression were performed under a CFX96^TM^ Real-Time PCR Detection System (Bio-Rad, Hercules, CA, United States) using the SYBR Green Real-Time PCR Master Mix Kit (TOYOBO, Osaka, Japan). The primers for RT-qPCR analysis are listed in **Table 1**. *Actin* and *UBQ5* genes were used as the housekeeping genes for RT-qPCR analysis, and the expression data were analyzed using the geometric mean of the two housekeeping genes.

### Measurement of Starch Concentration in Leaves

Leaves of 11-day-old Col-0 and *lbt* mutant grown on solid plates with 30 or 0.1 μMB were sampled at 10 a.m. Starch concentration was determined using the Starch Content Assay Kit (Solarbio, Beijing, China).

### Whole Genome Sequencing and Linkage Map Construction

Total genomic DNA of all plants was extracted from fresh leaves (Doyle, 1987). The whole genome sequence (WGS) of *lbt* mutant, Cs1909 and F_2_ population individuals were analyzed using an Illumina HiSeq^TM^ PE150 (Illumina Inc., San Diego, CA, United States). Ten *lbt* mutant plants and Cs1909 plants were sampled to construct two DNA-bulk for WGS, and the sequencing depth was 10×. The average sequencing depth of F_2_ population individuals was 1×. The homozygous single nucleotide polymorphisms (SNPs) between *lbt* mutant and Cs1909 were further structurally identified by alignment analysis with the reference genome (TAIR10^3^) using BWA software. The SNPs exhibiting sequencing errors or significant segregation distortion were filtered using a custom Perl script (Zhou et al., 2016). SNP index is calculated by the ratio between the number of reads of a mutant SNP and the total number of reads corresponding to the SNP. Homozygous SNPs were defined as SNPs with minimum coverage of the sites of three reads and a SNP index ≥ 0.9 (Abe et al., 2012). These SNPs markers were used to construct a high-density linkage map using JoinMap software version 4.0.

### Phenotypic Traits Evaluation and QTLs Analysis

To locate *LBT* gene, QTL mapping was performed. In this study, the primary root length of plants was used as the phenotype to identify QTLs for low-B tolerance. Under low-B condition, the line with long primary root length similar to *lbt* mutant was defined as low-B tolerance, vice versa, the line with short primary root length similar to Col-0 was defined as low-B sensitive. The F_2_ mapping population with 120 lines from a cross between *lbt* mutant and Cs1909 line was grown hydroponically with 30 μMB, and then, the F_2:3_ seeds were harvested from the individual F_2_ lines. Twenty F_2:3_ plants from each F_2_ line were grown on the solid media with low-B (0.1 μM) for 14 days, and the primary root length of the plants was determined. The average value of the primary root length of each F_2:3_ line was then used as the phenotype of F_2_ individuals to identify QTLs associated with low-B tolerance. Based on the constructed linkage map with the SNPs, the primary root length average values of each F_2:3_ were used for QTL analysis. QTL analysis with the composite interval mapping (CIM) method was conducted using model 6 of WinQTL cartographer 2.5 software (Ding et al., 2012) with an LOD threshold of 3.3.

### Statistical Analysis

All data were statistically analyzed using the software Statistical Product and Service Solutions 17.0 (SPSS, Chicago, IL, United States). Student’s *t*-test or Duncan’s multiple test was used to assess significant differences for *p* < 0.05.

## Results

### Effect of Low-B Stress on *lbt* Mutant Growth at the Seedling Stage

**FIGURE 1:**
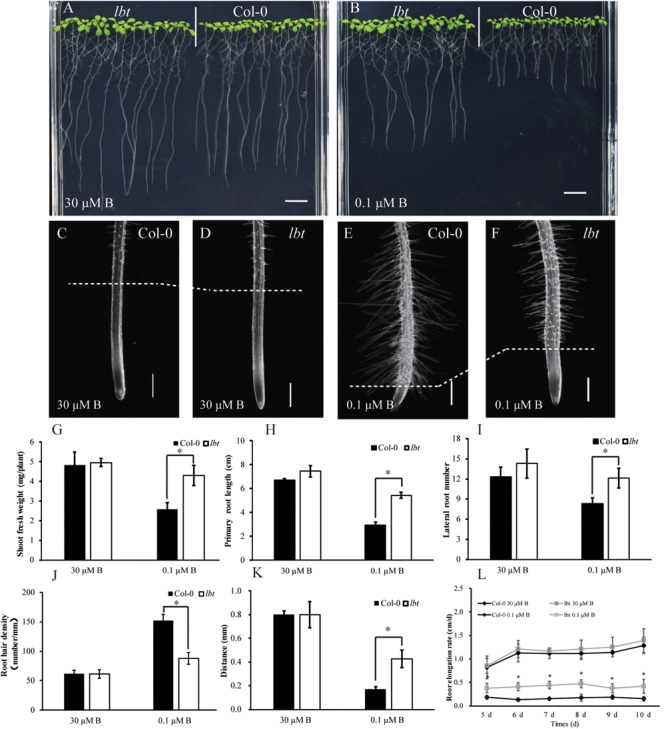
Performance of *Arabidopsis thaliana* wide-type Col-0 and *lbt* mutant. **(A,B)** Growth performance of Col-0 and *lbt* mutant grown on MGRL media for 7 days under 30 **(A)** and 0.1 **(B)** μMBconditions. Bars = 1 cm. **(C–F)** Stereoscopic microscopy analysis of Col-0 and *lbt* root tips under 30 **(C,D)** and 0.1 μMB **(E,F)** conditions. Bars = 200 μm. **(G,H)** Shoot fresh weight **(G)** and primary root length **(H)** of Col-0 and *lbt* mutant. Error bars indicate standard deviations of the mean (*n* = 14). **(I–L)** Lateral root number **(I)**, root hair density **(J)**, the distance from the root tip to the first appearance of root hairs **(K)** and primary root elongation rate **(L)** of Col-0 and *lbt* mutant. Error bars denote standard deviations of the mean (*n* = 5). **(G,H)** The asterisks show significant differences (Student’s *t*-test, *p* ≤ 0.05).

**FIGURE 2:**
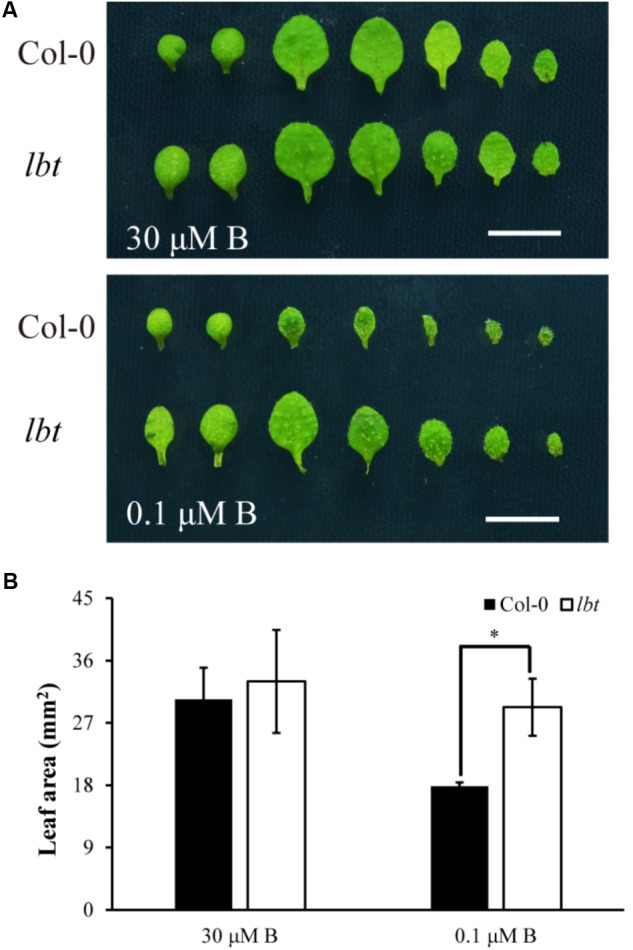
Leaf area of Col-0 and *lbt* mutant plants. **(A)** Leaf phenotype of Col-0 and *lbt* mutant seedlings under 30 μM and 0.1 μMB conditions for 11 days. On the left, two cotyledons precede the true leaves, and leaves are arranged from left to right in order of emergence. Bars = 5 mm. **(B)** Leaf areas of Col-0 and *lbt* mutant were measured. Error bars denote the standard deviations (*n* = 5). The asterisks indicate significant differences (Student’s *t*-test, *p* ≤ 0.05).

There was no significant difference in plant growth between Col-0 and *lbt* mutant at a normal B (30 μM) level (**Figure 1A**), but *lbt* mutant showed larger shoot and longer primary root length at a low-B (0.1 μM) level than Col-0 (**Figure 1B**). The fresh weight of *lbt* mutant was remarkably higher than that of Col-0 and the primary root length of *lbt* mutant was 85.5% longer than that of Col-0 under B deficiency conditions (**Figures 1G,H**). Both Col-0 and *lbt* mutant had similar root tip architecture at 30 μMB (**Figures 1C,D**), but *lbt* mutant showed more lateral roots, less root hair density and longer distance from the root tip to the first appearance of root hairs than that of Col-0 at 0.1 μMB condition (**Figures 1E,F,I–K**). The primary root length of both Col-0 and *lbt* mutant were recorded from the 5- to 11-day-old plants to estimate the root elongation rate. No significant differences in the root elongation rate were observed between Col-0 and *lbt* mutant at the 30 μMB condition. However, *lbt* mutant showed a faster root growth rate than that of Col-0 plants at the 0.1 μMB condition (**Figure 1L**). The leaf area of the 11-day-old *lbt* mutant was 64.1% larger than that of Col-0 at 0.1 μMB, but the difference was indistinguishable at the 30 μMB condition (**Figures 2A,B**). These results indicate that *lbt* mutant harbored high tolerance to low-B stress at the seedling stage.

### Effect of Low-B Stress on *lbt* Mutant Growth at the Reproductive Growth Stage

**FIGURE 3:**
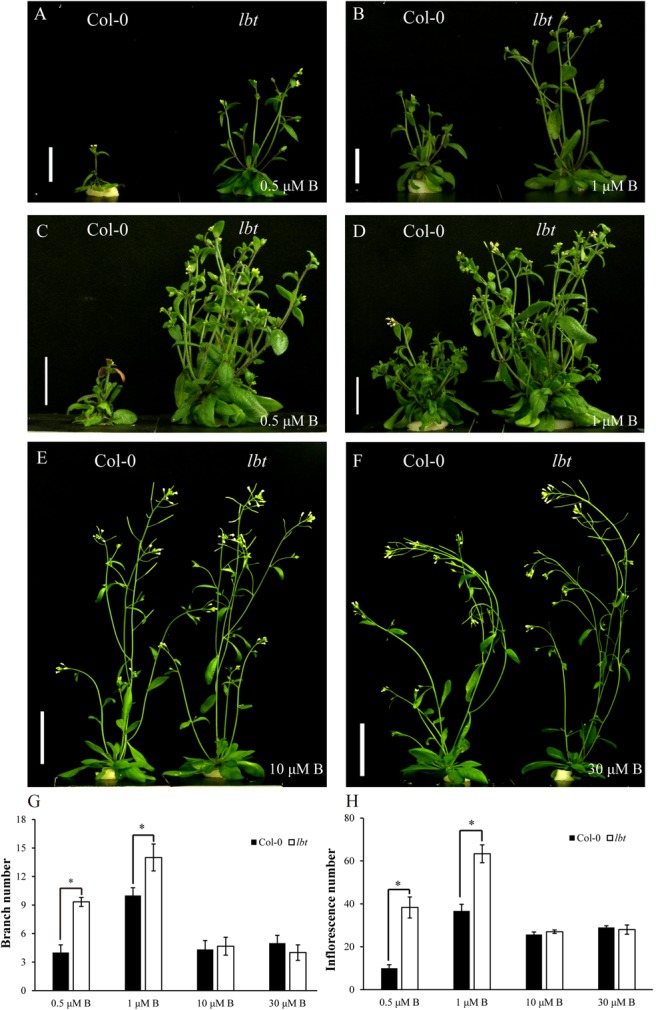
Reproductive growth of Col-0 and *lbt* mutant under different B conditions. **(A,B)** Col-0 and *lbt* mutant were grown for 35 days under 0.5 **(A)** and 1 **(B)** μMB conditions. Bars = 2 cm. **(C,D)** Col-0 and *lbt* mutant were grown for 45 days under 0.5 **(C)** and 1 **(D)** μMB conditions. Bars = 2 cm. **(E,F)** Col-0 and *lbt* mutant were grown for 35 days under 10 **(E)** and 30 **(F)** μMB conditions. Bars = 4 cm. **(G,H)** Branch and inflorescence number of Col-0 and *lbt* mutant under different B conditions. Branch number means the branch grown out from only the rosette base, and inflorescence number includes all the branches grown out from both the rosette base and branches (secondary branches and tertiary branches) from the first branches. Error bars denote the standard deviations (*n* = 3). The asterisks indicate significant differences (Student’s *t*-test, *p* ≤ 0.05).

To assess the differential responses to B deficiency between Col-0 and *lbt* mutant during the reproductive stage, the plants were grown in a hydroponic culture system. The shoot growth of Col-0 was inhibited severely relative to *lbt* mutant with B limitation (0.5 and 1 μM) for 35 days (**Figures 3A,B**). At these low-B conditions, the 45-day-old *lbt* mutant showed more branches and a greater inflorescences number than Col-0 (**Figures 3C,D,G,H**). However, *lbt* mutant grew similarly to Col-0 when a normal concentration of B was supplied (10 and 30 μMB, **Figures 3E,F**). Both genotypes were unable to produce seeds at 0.5 and 1 μM. *lbt* mutant was able to produce few seeds when it was cultured in the 2 μMB condition, whereas Col-0 did not produce any seeds in this condition (**Supplementary Figure S1**). These results indicate that *lbt* mutant exerted sustainable effect on the low B tolerance at both vegetative stage and reproductive stage.

### Effect of Other Nutrient Stresses on *lbt* Mutant Growth

**FIGURE 4:**
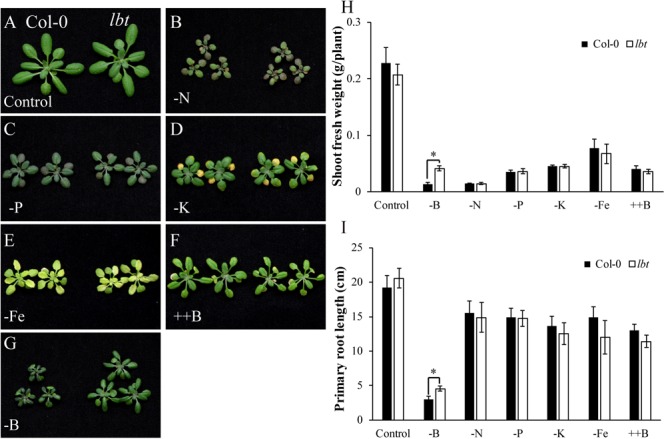
Responses of Col-0 and *lbt* mutant to different stresses. **(A–G)** Col-0 and *lbt* mutant grown in various stress conditions using a hydroponic culture system, including control **(A)**, –N (2 μM) **(B)**, –P (2 μM) **(C)**, –K (2 μM) **(D)**, –Fe (0.1 μM) **(E)**, ++B (3 mM) **(F)**, –B (0.1 μM) **(G)**. **(H,I)** Shoot fresh weight **(H)** and root length **(I)** of Col-0 and *lbt* mutant under various stresses. The asterisks show significant differences between Col-0 and *lbt* plants (Student’s *t*-test, *p* ≤ 0.05).

The low-B tolerance of *lbt* mutant led us to question whether *lbt* mutant has tolerance to other stresses. To clarify its tolerance, we cultured Col-0 and *lbt* mutant in diverse stresses using a hydroponic culture system including nitrogen (N) deficiency (-N, 2 μM), phosphorus (P) deficiency (-P, 2 μM), potassium (K) deficiency (-K, 2 μM), iron (Fe) deficiency (-Fe, 0.1 μM), and high B stress (++B, 3 mM). Similar to the result above, there was no significant difference in plant growth between Col-0 and *lbt* mutant for the normal control treatment (**Figure 4A**). N deficiency caused significant growth retardance and brownness of the cotyledons of both Col-0 and *lbt* mutant (**Figure 4B**). When the plants were cultured in the solution lacking P, the leaves gradually turned to dark green color (**Figure 4C**). Both Col-0 and *lbt* mutant were retarded with K deficiency, displaying strong yellow color in the cotyledons (**Figure 4D**). Most of the leaves of Col-0 and *lbt* mutant changed to yellow under Fe deficiency (**Figure 4E**). B toxicity led to necrosis of old leaf margin to comparable extent in both Col-0 and *lbt* mutant (**Figure 4F**). Distinctively, *lbt* mutant showed a larger shoot than Col-0 under B deficiency (**Figure 4G**). Taken together, there was no significant difference in phenotypical performance between Col-0 and *lbt* mutant under various nutrient deficiencies with the exception of B limitation, which was confirmed by the statistical analysis of shoot fresh weight and primary root length (**Figures 4H,I**). These results indicate that *lbt* mutant is specifically tolerant to B limitation.

### Expression of B Transport-Related Genes in *lbt* Mutant

**FIGURE 5:**
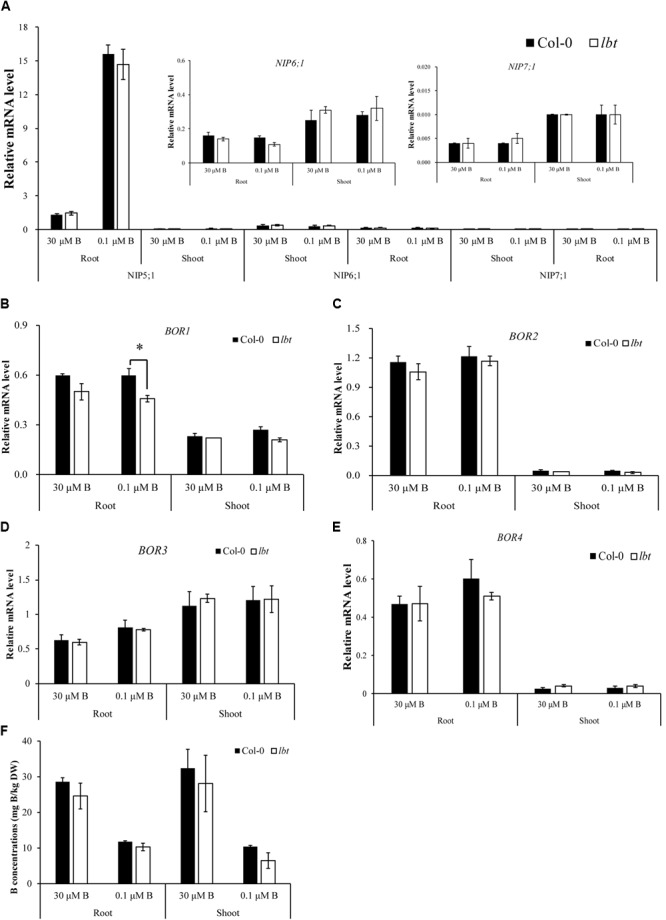
Relative expression of B transport-related genes in Col-0 and *lbt* mutant. **(A–F)** Relative expressions of *NIP5;1, NIP6;1* and *NIP7;1* **(A)**, *BOR1* **(B)**, *BOR2* **(C)**, *BOR3* **(D)**, *BOR4* **(E)** in root and shoot under 30 and 0.1 μMB. **(F)** B concentrations in root and shoot under 30 and 0.1 μMB. The expression data were analyzed using the geometric mean of two housekeeping genes *Actin* and *UBQ5*. Error bars denote the standard deviations (*n* = 3). The asterisks indicate significant differences (Student’s *t*-test, *p* ≤ 0.05).

It has been demonstrated that *NIP5;1, NIP6;1, BOR1* and *BOR2* are essential for B homeostasis under B limitation stress (Takano et al., 2002, 2006; Miwa et al., 2013). To elucidate whether the low-B tolerance of *lbt* mutant is mediated by B transport-related genes, we investigated the expression of *NIP5;1, NIP6;1, NIP7;1, BOR1, BOR2, BOR3*, and *BOR4* in *lbt* mutant by RT-qPCR. *NIP5;1* was up-regulated in roots by low-B without differences between Col-0 and *lbt* mutant (**Figure 5A**). However, the expression of *NIP6;1* and *NIP7;1* was significantly lower than *NIP5;1* without differences between Col-0 and *lbt* mutant in both root and shoot under 30 μMB or 0.1 μMB (**Figure 5A** and **Supplementary Figure S2**). The expression of *BOR1* was stronger in roots than in shoots regardless of B concentrations; however, *BOR1* mRNA abundance was lower in *lbt* mutant roots than in Col-0 root at the 0.1 μMB condition (**Figure 5B**). *BOR2* was insensitive to B concentrations but was strongly expressed in the roots of Col-0 and *lbt* mutant (**Figure 5C**). The expression of *BOR3* and *BOR4* in both root and shoot of Col-0 and *lbt* mutant was similar under 30 μMB or 0.1 μMB (**Figures 5D,E**).

Furthermore, we determined the B concentrations in both Col-0 and *lbt* mutant at the 30 and 0.1 μMB conditions (**Figure 5F**). The B concentrations of Col-0 and *lbt* mutant were higher under 30 μMB than under 0.1 μMB in the roots and shoots, but there was no significant difference in the B concentrations between Col-0 and *lbt* mutant. These results suggest that the low-B tolerance of *lbt* mutant might presumably not be related to the function of B transporters.

### Ultrastructure of *lbt* Mutant

**FIGURE 6:**
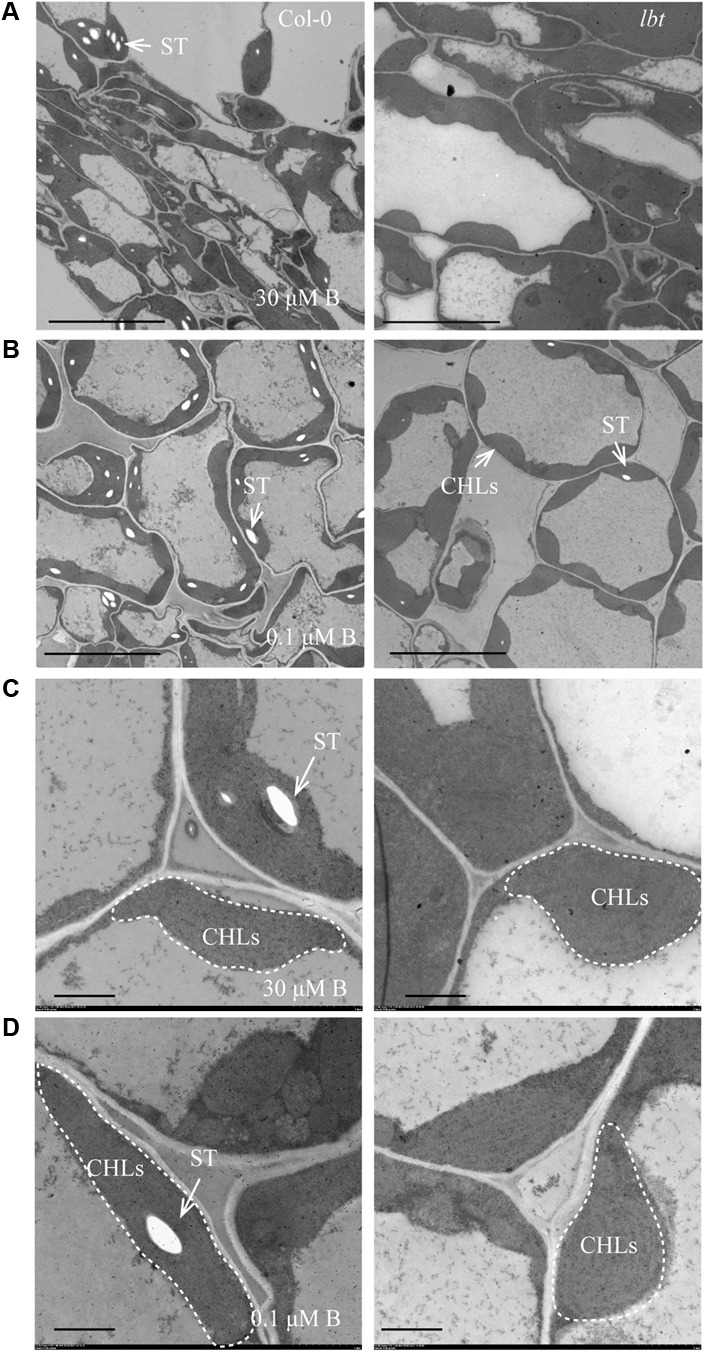
Transmission electron microscopy (TEM) analysis of the juvenile leaves of Col-0 and *lbt* 11-day-old seedlings. **(A,B)** The starch granules (ST) in chloroplasts (CHLs) in Col-0 and *lbt* mutant under 30 **(A)** and 0.1 **(B)** μMB conditions. Bars = 10 μm. **(C,D)** Local magnification of **(A,B)**. Bars = 2 μm.

To examine the differences in cell levels between Col-0 and *lbt* mutant, we performed the ultrastructure analysis of juvenile leaves of 11-day-old seedling using a transmission electron microscopy (TEM) system. Cells of both Col-0 and *lbt* mutant showed structural integrity, and the chloroplasts (CHLs) were orderly arrayed along the plasma membranes (PMs) at 30 μMB (**Figures 6A,C**) and 0.1 μMB (**Figures 6B,D**). Under the low-B condition more starch granules were accumulated and embedded in the chloroplasts of Col-0, however, tiny starch granules were observed in *lbt* mutant (**Figures 6B,D**).

### Relationship Between the Low-B Tolerance of *lbt* Mutant and T-DNA Insertion in At1g78860

**FIGURE 7:**
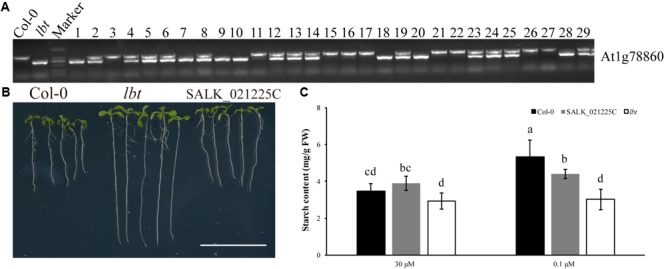
Genotype of F_2_ population with low-B tolerance phenotype. The primer is shown in **Table 1**. **(A)** Genotype of Col-0, *lbt* (At1g78860 T-DNA insertion) and 29 low-B tolerant individuals of the F_2_ population. *lbt* band is 1024 bp production of PCR with a T-DNA primer and gene-specific primer (At1g78860 Reverse) to detect T-DNA insertion. The WT band is 493 bp production of PCR with a pair of gene-specific primers (At1g78860 Forward and At1g78860 Reverse). **(B)** Phenotype of Col-0, *lbt* mutant (SALK_144144C) and SALK_021225C line under low-B condition. Bar = 2 cm. **(C)** Starch content of Col-0, SALK_021225C line and *lbt* mutant. Different letters indicate significant differences at *p*-value < 0.05.

To verify whether the low-B tolerance of *lbt* mutant was the result of T-DNA insertion in At1g78860, we constructed an F_2_ population by the cross of Col-0 and *lbt* mutant (T-DNA insertion line SALK_144144C of At1g78860, **Supplementary Figures S3A,B**) to analyze the co-segregation of the low-B tolerance phenotype with the T-DNA insertion genotype. Under the low-B (0.1 μM) condition, 29 F_2_ individuals with the low-B tolerance phenotype were chosen to identify their genotypes by PCR. The results indicate that 9 plants showed Col-0’s genotype with one band of 1024 bp, 7 plants were homozygous with one band (493 bp) of T-DNA insertion (*lbt*), and 13 plants were heterozygous among these 29 F_2_ plants (**Figure 7A**). This result indicates that low-B tolerance phenotype of these F_2_ population individuals was not co-segregated with their T-DNA insertion genotype, suggesting that the low-B tolerance of *lbt* mutant was not caused by T-DNA insertion in At1g78860.

To prove the results, another T-DNA insertion line of At1g78860 (SALK_021225C, **Supplementary Figures S3A,B**) was used to identify the phenotypes of fresh weight, primary root and starch accumulation under the low-B (0.1 μM) condition. There was no significant difference in these three traits between Col-0 and SALK_021225C line under normal and low-B conditions. Seven-day-old SALK_021225C line and Col-0 showed shorter primary root length than *lbt* mutant at 0.1 μMB condition (**Figure 7B**). The starch concentration of 11-day-old SALK_021225C line and Col-0 was significantly higher than that of *lbt* mutant under low-B condition (**Figure 7C**). These results indicate that SALK_021225C line is sensitive to low-B stress, like Col-0. Taken together, At1g78860 did not contribute to the low-B tolerance of *lbt* mutant.

### Mapping of QTL for Low-B Tolerance in *lbt* Mutant

To identify *LBT* gene in *A. thaliana*, genetic analysis of *lbt* mutant was carried out. A F_2_ population from Col-0 and *lbt* mutant was used to determine the segregation ratio of individual phenotypes. A total of 43 of 191 F_2_ individual phenotypes showed a tolerance phenotype similar to *lbt* mutant with long primary root length, and 148 F_2_ individuals showed a sensitive phenotype similar to Col-0 with short primary root length at the 0.1 μMB condition. The segregation ratios of short primary root length vs. long primary root length in the 191 F_2_ individuals was consistent with the expected Mendelian ratio of 3:1 (χ^2^ = 0.504; χ^2^ < χ^2^_0.01_ = 6.64, **Table 2**). This result indicates that the low-B tolerance achieved by *lbt* mutant is controlled by a monogenic recessive mutation.

**Table 2:** The segregation ratio of low-B sensitive lines to low-B tolerance lines in F_2_ population.

	Parents		F_2_ population			
	**Col-0**	***lbt* mutant**	**Line with short PRL^a^**	**Line with long PRL**	**Ratio of shoot PRL to long PRL**	**χ^2^**

PRL (mean ± SD^b^, cm)	2.62 ± 0.28	6.12 ± 0.28	2.44 ± 0.34	6.23 ± 0.73		
Line number	10	10	148	43	3.44:1	0.504

*^*a*^PRL, primary root length. ^*b*^SD, standard deviation. The F_2_ was developed from a cross between Col-0 and lbt mutant and the segregation ratio fitted the expected Mendelian ratio of 3:1 in the F_2_ population (χ^2^ < χ^2^_0.01_ = 6.64).*

**FIGURE 8:**
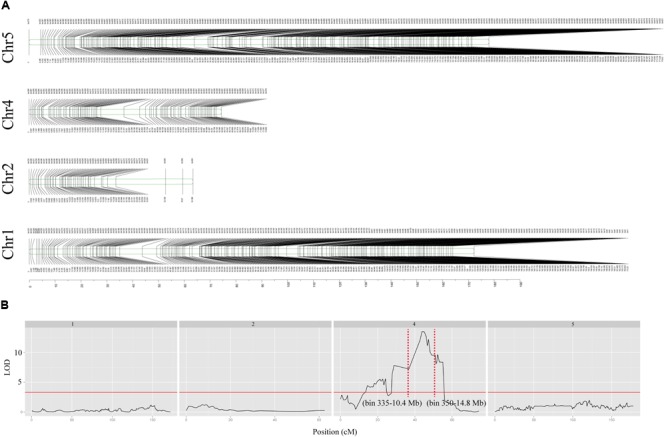
Linkage genetic map and positions of putative QTL. **(A)** Linkage genetic map of chromosomes 1, 2, 4, and 5 were constructed with 620 SNPs bin markers. Marker names are shown above each chromosome and map distances (cM) are shown below each chromosome. **(B)** Mapping of QTL for low-B tolerance on each chromosome, the *y*-coordinate represents the LOD value and the *x*-coordinate represents the genetic location. The red solid line indicates an LOD threshold of 3.3, and the red dashed line refers to the QTL flank with bin marker.

The F_2_ mapping population with 120 individuals derived from a cross of Cs1909 × *lbt* were sequenced together with the parents based on WGS technique. A total of 102,409 homozygous SNPs were identified by the WGS analysis of the two parental lines. The SNPs that exhibited significant segregation distortion in the F_2_ population were filtered out (*p* < 0.001, χ^2^ test) and then 102,283 SNPs were selected from the F_2_ population, which were divided into 620 bin markers for linkage genetic map construction using JoinMap 4.0 software. As a result, these bin markers were assigned to 4 chromosomes (**Figure 8A**). Based on the high-density linkage genetic map of 620 bin markers, the primary root length (**Supplementary Figure S4**) under the low-B condition of the F_2_ population were used for QTL mapping. One QTL for low-B tolerance was identified and flanked by two bin markers (bin 335-bin 350), and it was physically located in the region of 10.4–14.8 Mb on chromosome 4 (**Figure 8B**). This QTL could explain 75.1% of the phenotypic variance of low-B tolerance, and the LOD value was as high as 12.2 (**Figure 8B**). A total of 1,324 genes existed in this QTL interval, but it does not contain any reported B-related genes.

## Discussion

In the present study, we reported an *A. thaliana* mutant *lbt* that showed high tolerance to B deficiency. This low-B tolerant phenotype was accompanied by improved growth performance under low-B conditions (**Figure 1**). The QTL for low-B tolerance was then mapped to chromosome 4 by genetic analysis.

### Identification of a Low-B Tolerance Specific Mutant *lbt* With Low B Demand

Root growth was more sensitive to B limitation than shoot growth in *A. thaliana* (Takano et al., 2001). Our research indicates that *lbt* mutant was overly different from Col-0 in both shoot and root growth under B deficiency. *lbt* mutant showed improved growth performance during the vegetative stage and reproductive stage under B limitation stress (**Figures 1–3**). Most importantly, *lbt* mutant showed low-B tolerance specificity (**Figure 4**). Unexpectedly, the B concentrations in the root and shoot of *lbt* mutant were indistinguishable from those of Col-0 under low-B conditions (**Figure 5F**), suggesting that *lbt* mutant has low B demand under low-B conditions. To the best of our knowledge, at present, no research studies have reported the identification of low B demand line. The overexpression of tonoplast-localized *TIP5;1* in *A. thaliana* conferred tolerance to B toxicity without a B concentration change in the plants (Pang et al., 2010). *AtTIP5;1* accumulates on the tonoplast, facilitating the influx of B in vacuoles and leading to the compartmentalization of B. Similarly, overexpression of *DUR3* gene in yeast slightly enhanced its sensitivity to B toxicity but did not affect the cellular B concentration (Nozawa et al., 2006). Taken together, it is an important finding that *lbt* mutant has low-B tolerance specificity with low B demand in our study.

### *lbt* Mutant Tolerant to Low-B Condition Is Independent on Differential Expression of B Transporter Genes

Many genes involved in the uptake and transport of B have cloned from *A. thaliana*, including members of the NIP family and BOR family (Takano et al., 2002, 2006; Miwa et al., 2013). The B concentrations of both the root and shoot of the *nip5;1* mutants and the leaves of the *bor1* mutants were lower than that of wild-type plants at B limitation (Noguchi et al., 1997; Takano et al., 2006). This difference is due to the loss of uptake function in the *nip5;1* mutant and translocation function in *bor1* mutants, respectively. In our study, there was no significant difference in the expression of *NIP5;1* and *BOR2* genes between Col-0 and *lbt* mutant at both normal and low-B conditions (**Figures 5A,C**). Furthermore, compared to Col-0, *BOR1* transcription abundance decreased at low-B conditions, but this decrease cannot account for the growth improvements of the shoots in *lbt* mutant (**Figure 5B**). These results suggest that transporter-related genes are not the determinant of low-B tolerance in *lbt* mutant.

### *lbt* Mutant With Less Starch Accumulation Compared With Col-0 Under Low-B Condition

Previous studies have shown that B deficiency widely caused starch granule accumulation in plants, e.g., tobacco leaves (Camacho-Cristóbal et al., 2004), citrus leaves (Han et al., 2008) and navel orange leaves (Liu et al., 2015). Starch synthesis is ascribed to a cascade of enzyme functions in pathways from the Calvin–Benson cycle (Guo et al., 2017), and B deficiency inhibits plant photosynthetic capacity (Kastori et al., 1995; Zhao and Oosterhuis, 2002). These authors thus speculated the possible mechanisms that are responsible for B deficiency triggered starch accumulation in plants, such as plants reduced the ability to translocate photosynthates, decreased the demand for carbon in growing tissues due to growth inhibition or decreased the sugar usage under B deficiency. In our study, TEM analysis at the cell level revealed the distinct starch accumulation in juvenile leaves between Col-0 and *lbt* mutant (**Figure 6**). Consistent with this observation, starch level assay showed Col-0 leaves had significantly higher starch level at 0.1 μMB than at 30 μMB, whereas *lbt* mutant accumulated steady state level of starch which is significantly lower than Col-0 at 0.1 μMB (**Figure 7C**). Based on these results, we propose that the accumulation of the starch in Col-0 is merely a consequence of a B-deficiency-induced growth inhibition because less carbon demand is fulfilled. In contrast, the less growth inhibition of *lbt* mutant under B deficiency brings about sugar demand increase which reasonably contributes to starch consumption to some extent. We yet consider another possibility by which the starch synthesis was inhibited or its degradation was facilitated in *lbt* mutant due to the mutation of *LBT* gene. Starch can be degraded, which then generates plentiful sugars, such as maltose, glucose, and glucose-1-phosphate via enzymatic steps for energy consumption (Stitt and Zeeman, 2012). The accumulated precursors (e.g., Glc-6-P, Glc-1-P, and UDPG1c) for starch synthesis can be directly used rapidly growing tissues, decreasing starch accumulation. The mechanisms understanding low-B tolerance through the pathway of starch metabolism in *lbt* mutant require further experimental evidence on the precise cloning of *LBT* gene.

In this study, we found that T-DNA insertion was not relevant to the phenotype of *lbt* mutant, and the tolerance of *lbt* mutant to low-B stress was also not related to B transport-related genes. These results suggest that the insertion of T-DNA caused other mutations in the genome and affected the function of a gene with unknown function, which led to the phenotypic changes. To locate the target gene, we identified one major QTL for low-B tolerance, indicating that the QTL is located on chromosome 4 between 10.4 and 14.8 Mb, which contains 1324 genes, such as protein kinase genes, F-box family genes, UDP-galactose transporters and a large number of genes with unknown function. However, there are no reported B-related genes in this region. In future work, a larger F_2_ population based on a cross of *lbt* mutant and Cs1909 line and fine-mapping is required to precisely identify *LBT* gene in the genomic region, which may contribute to a novel understanding on the mechanism of low-B tolerance and breeding research with the cloning and function analysis of *LBT* gene.

## Author Contributions

ZH and FX designed the experiments. ZH and LP conducted the experiments. ZH, SW, and FX analyzed the data and wrote the article. HZ and LS provided technical guidance and revised the article.

## Conflict of Interest Statement

The authors declare that the research was conducted in the absence of any commercial or financial relationships that could be construed as a potential conflict of interest.

## Footnotes

1 http://abrc.osu.edu/

2 http://rsb.info.nih.gov/ij/

3 ftp://ftp.ensemblgenomes.org/pub/plants/release-34/fasta/arabidopsis_thaliana/dna/

## Supplementary Material

FIGURE S1 Reproductive growth of Col-0 and *lbt* mutant under 2 μMB for 60 days conditions. **(A)** The first branch of Col-0 and *lbt* mutant. Bars = 2 cm. **(B)** The pod of Col-0 and *lbt* mutant. Bars = 2 mm.

FIGURE S2 Expressions of *NIP5;1, NIP6;1* and *NIP7;1* genes. Transcripts of the *NIP5;1, NIP6;1* and *NIP7;1* genes were detected by RT-PCR.

FIGURE S3 **(A)** Structure of At1g78860 gene. Black box represents exon, gray boxes represent 5′UTR and 3′UTR and bars indicate other genomic regions. Triangles indicate the T-DNA insertions. **(B)** Relative expression of At1g78860 in Col-0, SALK_021225C and *lbt* mutant. Different letters indicate significant differences at *p*-value < 0.05.

FIGURE S4 The primary root length of Cs1909, *lbt* mutant and F_2_ population individuals.
