# Supplementary figures and images for: Identification and Characterization of an Arabidopsis thaliana Mutant lbt With High Tolerance to Boron Deficiency
Source: Front Plant Sci. 2018 Jun 4;9:736. doi: 10.3389/fpls.2018.00736 (PMC5994474; doi:10.3389/fpls.2018.00736)

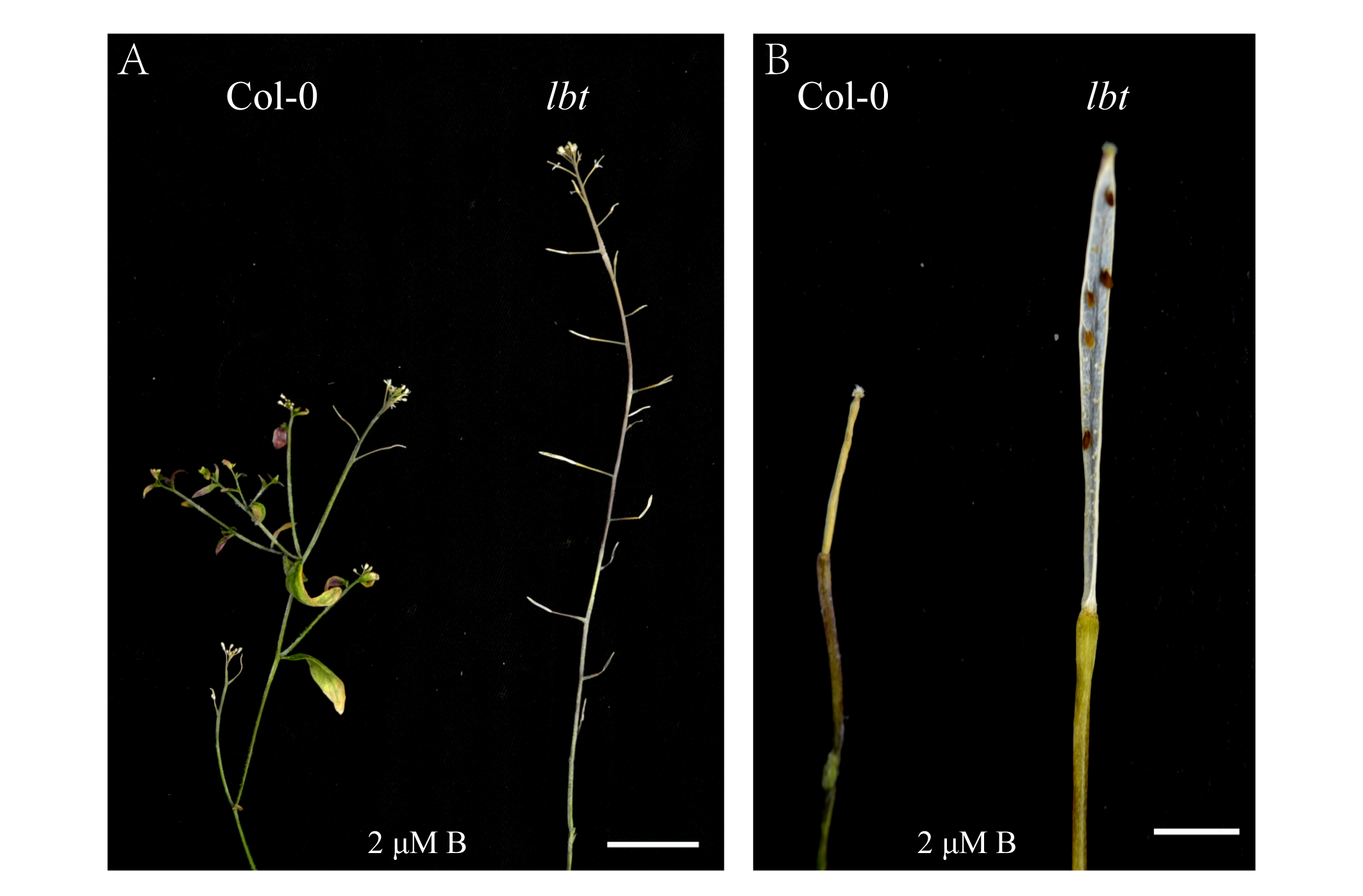

Supplement: FIGURE S1 — Reproductive growth of Col-0 and lbt mutant under 2 μMB for 60 days conditions. (A) The first branch of Col-0 and lbt mutant. Bars = 2 cm. (B) The pod of Col-0 and lbt mutant. Bars = 2 mm. [file Image_1.TIF]

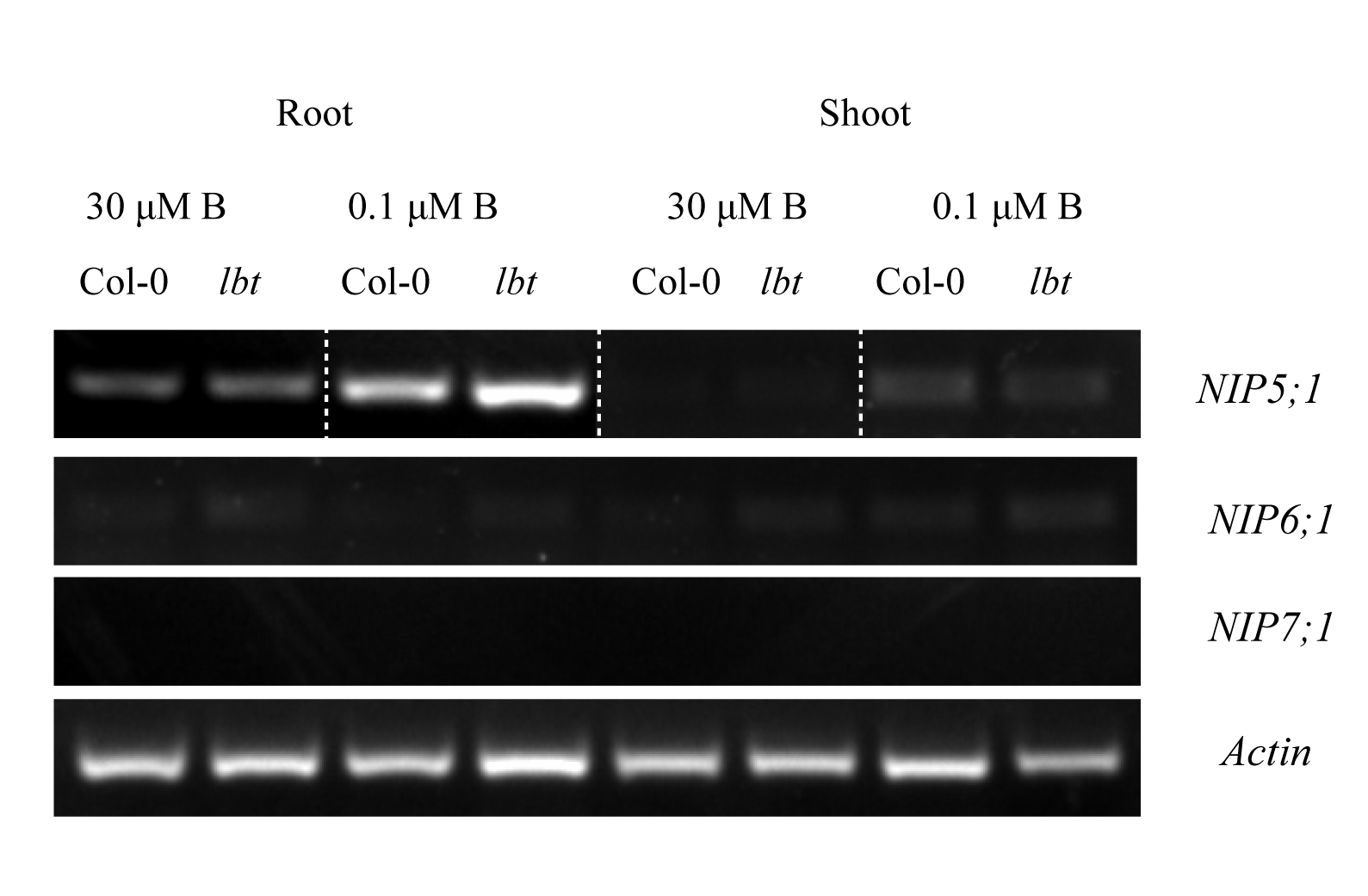

Supplement: FIGURE S2 — Expressions of NIP5;1, NIP6;1 and NIP7;1 genes. Transcripts of the NIP5;1, NIP6;1 and NIP7;1 genes were detected by RT-PCR. [file Image_2.TIF]

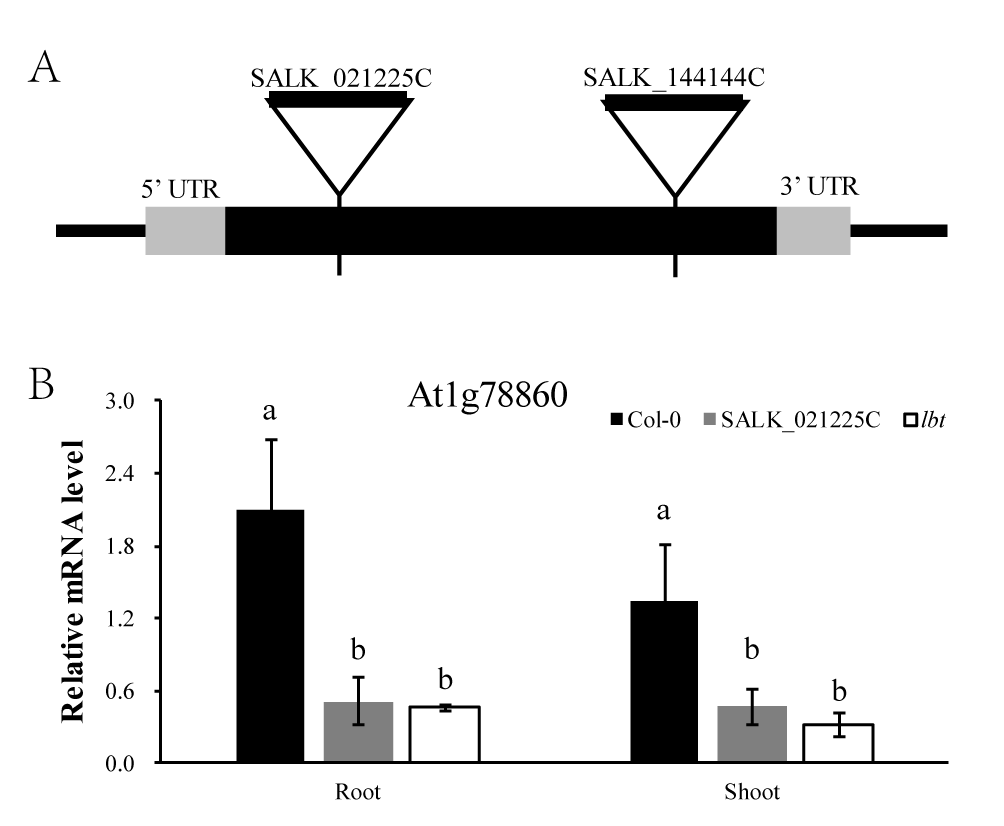

Supplement: FIGURE S3 — (A) Structure of At1g78860 gene. Black box represents exon, gray boxes represent 5′UTR and 3′UTR and bars indicate other genomic regions. Triangles indicate the T-DNA insertions. (B) Relative expression of At1g78860 in Col-0, SALK_021225C and lbt mutant. Different letters indicate significant differences at p-value < 0.05. [file Image_3.TIF]

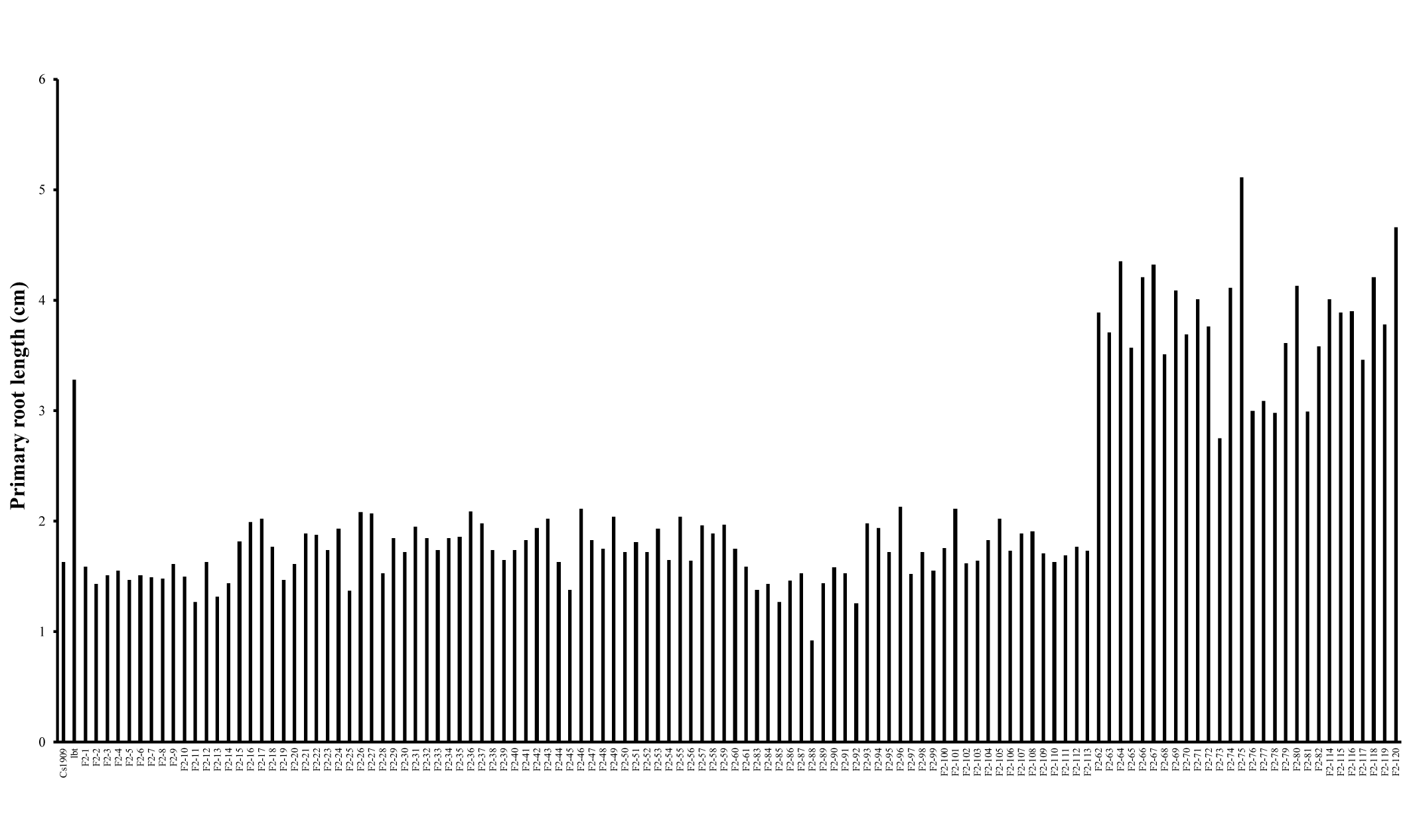

Supplement: FIGURE S4 — The primary root length of Cs1909, lbt mutant and F2 population individuals. [file Image_4.TIF]
